# Neuroprotective Treatments for Digestive Forms of Chagas Disease in Experimental Models: A Systematic Review

**DOI:** 10.1155/2022/9397290

**Published:** 2022-09-25

**Authors:** José Rodrigues do Carmo Neto, Rhanoica Oliveira Guerra, Wellington Francisco Rodrigues, Marcos Vinicius da Silva, Juliana Reis Machado

**Affiliations:** ^1^Department of Bioscience and Technology, Institute of Tropical Pathology and Public Health, Federal University of Goias, Goiânia, Goiás, Brazil; ^2^Department of Microbiology, Immunology and Parasitology, Institute of Biological and Natural Sciences of Federal University of Triângulo Mineiro, Uberaba, Minas Gerais, Brazil; ^3^Postgraduate Course in Health Sciences, Federal University of Triângulo Mineiro, Uberaba, Minas Gerais, Brazil; ^4^Department of General Pathology, Federal University of Triângulo Mineiro, Uberaba, Minas Gerais, Brazil

## Abstract

Chagas disease is an anthropozoonosis caused by the protozoan *Trypanosoma cruzi* and is characterized as a neglected disease. It is currently endemic in 21 countries on the Latin American continent, including Bolivia, Argentina, and Paraguay. Unfortunately, there are no optimally effective treatments that can reduce the damage caused in the digestive form of the disease, such as the neuronal destruction of the myenteric plexus of both the esophagus and the colon. Therefore, the objective of this systematic review was to report the possible pharmacological neuroprotective agents that were tested in murine models of the digestive form of Chagas disease. Inclusion criteria are *in vivo* experimental studies that used different murine models for digestive forms of Chagas disease related to pharmacological interventions with neuroprotective potential, without year and language restriction. On the other hand, the exclusion criteria were studies that did not approach murine models with the digestive form of the disease or did not use neuroprotective treatments, among others. The search in the PubMed, Web of Science, Embase, and LILACS databases was performed on September 4, 2021. In addition, a manual search was performed using the references of the included articles. The risk of bias assessment of the studies was performed based on the SYRCLE tool guidelines, and the data from the selected articles are presented in this review as a narrative description and in tables. Eight articles were included, 4 of which addressed treatment with acetylsalicylic acid, 3 with cyclophosphamide, and 1 with Lycopodium clavatum 13c. In view of the results of the studies, most of them show neuroprotective activity of the treatments, with the potential to reduce the number of damaged neurons, as well as positive changes in the structure of these cells. However, more studies are needed to understand the mechanisms triggered by each drug, as well as their safety and immunogenicity. Systematic review registration is as follows: PROSPERO database (CRD42022289746).

## 1. Introduction

Chagas disease (CD), caused by the flagellate protozoan *Trypanosoma cruzi*, represents a neglected disease that affects 8 to 11 million people worldwide. In general, the disease can be divided into two phases: acute with nonspecific symptoms in most cases and chronic, which can be symptomatic or asymptomatic [1]. In the symptomatic chronic phase, the disease is related to the development of cardiac and/or digestive tract changes (megaesophagus and/or megacolon). Digestive forms comprise up to 10-21% of symptomatic CD cases, with megaesophagus having the highest incidence, followed by megacolon [2]. Unfortunately, there is a lack of effective treatments at this stage [3].

Although the pathogenesis of the digestive forms is not so clear, it is suggested that the inflammation induced by the infection is one of the essential points for the progression of the disease, mainly because it affects neurons of the myenteric plexus in both the esophagus and the colon [4]. Studies report that *T. cruzi* infection in experimental models induces neuronal destruction, starting in the acute phase. Thus, it is suggested that immune system components such as macrophages, NK cells, eosinophils [5], nitric oxide (NO) and IFN-*γ* play a role in neuronal destruction [6–9]. In addition, homeostasis-related components of the enteric nervous system have also been reported to be altered after infection.

While neuronal destruction occurs, processes such as neuronal hypertrophy or atrophy increase in the wall of the esophagus and colon and in the muscular layers of these organs which have also been reported. With the passage of time, all these processes culminate in the alteration of the functioning of the organ, loss of peristalsis, and, consequently, the stoppage of the passage of food or fecal bolus.

Isosorbide and nifedipine are drugs tested in clinical trials and used for the treatment of megaesophagus in humans, with the aim of improving the passage of food through the organ [10–14]. Although the use of isosorbide has shown lower rates of esophageal retention and severity of dysphagia, few studies have actually evaluated the impact of these treatments [15]. In addition, side effects such as headache are common during treatment with isosorbide, which decreases patients' adherence to therapy [16]. Pneumatic dilatation or surgery is also indicated depending on the stage of the megaesophagus. For chagasic megacolon, changes in life habits, such as diets rich in fiber and high-water intake, are indicated. Pharmacologically, laxatives are also used. Surgical interventions are only used in severe cases, such as severe refractory constipation and other complications [2]. Thus, most treatments for CD help with the symptoms of digestive forms and can be invasive.

Due to this problem, there is a need to develop new alternatives for the treatment of digestive forms that aim to destroy the parasite and reduce the inflammatory response and consequently neuronal protection. Therefore, the purpose of this systematic review is to report the possible pharmacological neuroprotective agents that were tested in experimental animal models for CD, in its digestive form.

## 2. Methods

### 2.1. Protocol and Record of the Systematic Review

The present systematic review was conducted in accordance with the methodological guidelines proposed by the Key Items for Reporting Systematic Reviews and Meta-analyses (PRISMA) [17]. The protocol of this review was registered in the database called PROSPERO (International Prospective Register of Systematic Reviews), with registration number CRD42022289746.

### 2.2. Eligibility Criteria

The development of the systematic review in question was based on a guiding question: “Are there pharmacological interventions that prevent neuronal loss in the myenteric plexus in digestive forms of CD in experimental murine models?”. Thus, for the assembly of the search strategy and the establishment of eligibility criteria, the acronym model PICOT (population, intervention, comparator, outcome, and types of studies) was used:

P: murine models of digestive Chagas disease

I: pharmacological treatment

C: no treatment (control group)

O: neuroprotection

T: *in vivo* studies

Therefore, only experimental *in vivo* studies were included, which used different experimental models for digestive forms of CD related to pharmacological interventions with neuroprotective potential. For the exclusion criteria, the following points were followed:

1. Not murine models of digestive forms of Chagas disease
2. Not pharmacological treatment with neuroprotective potential in acute or/and chronic phases of Chagas disease
3. Studies that focus on treatment and do not assess neuronal counts
4. Studies that do not use pharmacological treatment and evaluate neuronal counts (example: effect of physical activity and neuronal protection)
5. Studies that do not compare infected and treated animals with infected and untreated animals
6. Letter to the editor, editorial, conference documents, commentary, news, descriptive and systematic reviews, and book chapters
7. Any measurement that does not show a biological effect

### 2.3. Sources of Information and Search

According to the indications of the Peer Review of Electronic Search Strategies (PRESS) [18], the search strategy was developed and submitted for evaluation by a subject specialist. For setting up the strategy (presented in supplementary materials S1), the PubMed database was considered as the standard, and year of publication and language were not considered as exclusion factors.

To carry out the bibliographic search, four research bases were used: PubMed, Web of Science, Embase, and LILACS. The details of the searches in each database are exposed in supplementary materials. In addition to these bases, manual searches focused on the reference list of the included articles were performed. After the search was completed, duplicate articles were tracked and removed using the EndNote X9® program.

### 2.4. Selection of Studies and Data Extraction

The first step of article selection was performed by two evaluators (J.R.C.N and R.O.G) independently and blindly. The titles and abstracts of all articles obtained through the search were evaluated for inclusion or exclusion. To assist in this step, the Rayyan–Intelligent Systematic Review program was used. After analyzing the articles, possible disagreements between the reviewers were agreed upon in a discussion between them.

In the second step, the articles selected in the first step were transferred to an Excel table with the following information: authors and year, article title, inclusion or exclusion, final status, and justification for exclusion. Then, only articles focusing on the use of some intervention with neuroprotective potential in murine models for digestive forms of CD were included. All those who did not meet the inclusion criteria were excluded. The reviewers performed this step blindly and independently (J.R.C.N and R.O.G). In addition, possible disagreements between the reviewers were agreed upon in discussion between them.

Relevant data were extracted from all included studies by two independent evaluators (J.R.C.N and R.O.G). Thus, the information collected in the *in vivo* studies was as follows: intervention used, experimental model, groups evaluated and number of animals used, strain used, route of infection/inoculum used, evaluation phases, form of induction of the chronic phase, treatment regimen, concentration of the intervention used/treatment route, mortality rate, organ and region evaluated, methodology used for neuronal analysis/analyzed region, number of fields and neurons analyzed, number of neurons per group, area of the neuronal body per group (*μ*m^2^ or cm^2^), neuronal cytoplasm area per group (*μ*m^2^), neuronal nucleus area per group (*μ*m^2^), other observed biological phenomena, and reference.

The WebPlotDigitizer tool was used to obtain apparently hidden data in the article. Through this tool, it was possible to extract values present only in graphs.

### 2.5. Risk of Bias in *In Vivo* Studies

To assess the risk of bias, the Systematic Review Center for Laboratory Animal Experimentation (SYRCLE) tool was used [19]. This step was also performed by two reviewers independently (J.R.C.N and R.O.G). Possible disagreements between the reviewers were agreed upon in discussion between them. The tool consists of six categories: selection bias, performance bias, detection bias, attrition bias, reporting bias, and other sources of bias. Each category had some questions, which are exposed in supplementary materials, totaling 10 to help reviewers classify each article included. For each question, it was necessary to answer “yes,” “no,” or “uncertain,” with each of these judgments corresponding to a color: red, green, or yellow, respectively. The 10 questions used for the in vivo risk of bias assessment are listed in Supplemental Materials S2.

### 2.6. Synthesis Methods

The main findings of the studies were presented through a narrative description, and, whenever possible, a comparison between them was performed. In addition, data from the articles (in topic 2.4) were tabulated (Table 1). Statistical analyses such as meta-analysis, heterogeneity, and sensitivity analyses were not applied.

## 3. Results

### 3.1. Search for PRISMA Studies and Flowchart

**Figure 1:**
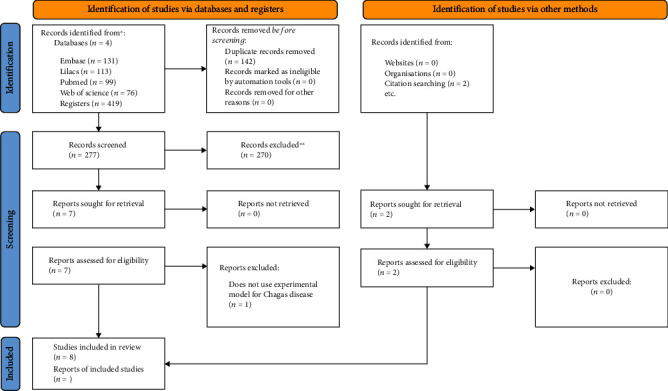
PRISMA flow chart of the study selection and inclusion process. PRISMA: Preferred Reporting Items for Systematic Reviews and Meta-analyses.

The search in the databases for studies that evaluate different neuroprotective interventions for digestive forms of CD in experimental models resulted in 419 articles. In addition to this amount, 2 articles were obtained from a manual search through the reference list of articles, totaling 421. In the duplicate article tracking step, 142 articles were excluded, with a total of 279 for analysis. Then, analysis by title and abstract was performed, totaling 9 potential articles included and 270 excluded. Of those included in the previous step, the full articles were read and only 8 studies were included, with the exclusion of 1. The exclusion of the article was based on the nonuse of an experimental model for Chagas disease. Thus, 8 articles were considered eligible and followed with the qualitative analysis (Figure 1). The list of screened articles and the final status of each are listed in the Supplemental Materials S3.

### 3.2. Study Characteristics

The first article focusing on neuroprotective interventions for the digestive forms of CD in experimental models was published in 2006. As of that year, 2017 was the year with the highest number of publications (3), representing 37.5% of articles. The last article published on the topic was in 2019.

**Figure 2:**
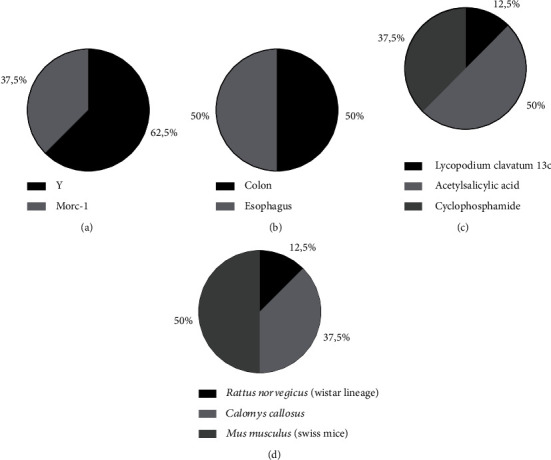
General characteristics of studies included in the systematic review (*n* = 8). List of *Trypanosoma cruzi* strains used (a), as well as organs used to study the digestive form of Chagas disease (b). In addition, there is also a list of pharmacological interventions (c) and the experimental models used (d).

To conduct the experiments, only two strains were used to infect the animals: strain Y (5-62.5) and Morc-1 (3-37.5%) (Figure 2(a)). Finally, the colon (4) and esophagus (4) were evaluated in the same number of articles (Figure 2(b)).

Three different interventions were used in the included articles: acetylsalicylic acid (ASA) (4), cyclophosphamide (3), and Lycopodium clavatum 13c (LC) (1). Thus, ASA is the most focused intervention in the studies, with 50%, followed by cyclophosphamide, with 37.5% and LC with 12.5% (Figure 2(c)).

Furthermore, to assess the impact of each intervention, three experimental models were approached: *Mus musculus* (Swiss mice) (4), *Calomys callosus* (3), and *Rattus norvegicus* (Wistar lineage) (1). Swiss mice represented the most used model in 50% of the articles, followed by *Calomys callosus* (37.5%) and *Rattus norvegicus* (Wistar lineage) (12.5%) (Figure 2(d)).

### 3.3. Risk of Bias Assessment of *In Vivo* Studies

**Figure 3:**
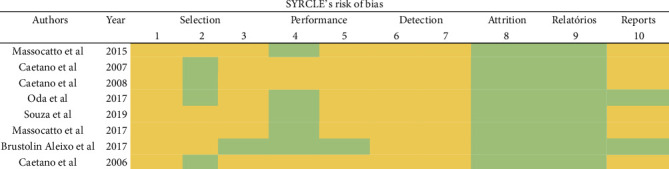
Risk of bias assessment of *in vivo* studies. Prepared based on the SYRCLE tool [19]. Green (low risk of bias), red (high risk of bias), and yellow (uncertain risk of bias).

For the assessment of risk of bias, all 8 articles included were analyzed. As shown in Figure 3, most articles did not clearly address the selection bias criteria (items 1, 2, and 3), detection (items 6 and 7), and others (item 10). Within these items, the lack of exposure and reporting of allocation criteria, baseline characteristics such as initial animal weight, allocation concealment, random housing, blinding of caregivers and outcome assessors, and randomization of animals were unclear. Finally, 75% of the selected works did not declare information regarding a possible conflict of interest.

### 3.4. Effects of Interventions on Experimental Models

The effects of the interventions were subdivided according to the type of intervention in each study, they are arranged below, and the main information is summarized in Table 1.

#### 3.4.1. Lycopodium Clavatum 13c (LC)

Among the eight articles, LC was evaluated in only one study [20].

To analyze the impact of LC, *Rattus norvegicus* of the Wistar strain were infected with the Y strain of *T. cruzi*. The treatment induced a predominant proinflammatory profile at the beginning of infection, at 10-day postinfection, with an increase in IFN-*γ* and at 24 days with an increase in serum IL-12. Interestingly, at 24 days, anti-inflammatory cytokines (IL-10 and IL-4) were also found to be increased in the treated group compared to the untreated group, which demonstrates a balance between proinflammatory and anti-inflammatory/regulatory responses. Regarding the number of neurons in the distal colon at 322-day postinfection, the authors observed that the treatment induced neuronal protection when compared to the untreated group. Furthermore, the use of LC induced maintenance of the number of these cells, while in the untreated group, there was a reduction along the 125 × 322-day postinfection. LC treatment also induced hypertrophy in neurons present in the distal and proximal colon 125-day postinfection, but after 322 days of infection, this effect was observed only in the distal colon. Thus, an increase in the body area, cytoplasm, and nucleus of neurons was reported when compared to the untreated group [20].

Although the authors demonstrate that LC induces neuronal protection and even suggest that this protection is mediated by the establishment of a treatment-mediated immune balance profile, there is a lack of an uninfected control to further refine the comparisons between the experimental groups [20].

#### 3.4.2. Cyclophosphamide

Among the eight articles, cyclophosphamide was evaluated in three studies, one focused on the impact of treatment on the colon [21] and the other two on the esophagus [22, 23].

All studies showed that cyclophosphamide treatment induces an increase in parasitaemia when compared to the infected and untreated group, especially after 10 days of infection. Furthermore, the use of cyclophosphamide reduced the production of NO in exudate from peritoneal macrophages from young (10 days of infection) and old mice (450 days of infection). The intervention also acted on the proliferation of splenocytes, reducing the proliferative capacity of these cells when exposed to polyclonal stimuli. Regarding neuronal count, the treatment resulted in protection of these cells both in the esophagus [22, 23] and in the colon [21] in both phases evaluated (10 days of infection and 450 days of infection). Regarding morphometric analyses of esophageal neurons (diameter, perimeter, area, and volume), treatment with cyclophosphamide did not induce any changes when the treated and infected groups were compared with the untreated infected group [22, 23]. However, older animals showed lower values in all parameters analyzed when compared to younger ones [23]. For colon neurons [21], the treatment did not induce morphometric changes at 10 days of infection. Only at 450 days of infection, it was observed that the use of cyclophosphamide increased the perimeter, area, and volume of neurons when compared to the respective untreated group.

#### 3.4.3. Acetylsalicylic Acid (ASA)

Among the eight articles, ASA was evaluated in four studies, two focused on the esophagus [24, 25], and two on the colon [26, 27].

The first study that used ASA for Chagas disease aimed to assess the impact of this intervention on esophageal nitrergic neurons in an experimental model of chronic phase [24]. For this, Swiss mice were infected with the Y strain. When evaluating the total parasitaemia, it was reported that treated mice showed an increase of 13.52% in this parameter. Although infection increases the neuronal nitrergic population and this increase is maintained in the infected and treated groups, the use of ASA did not result in neuronal protection or destruction. However, the intervention was shown to prevent infection-induced atrophy in 20.33% of neurons by increasing the nuclear (17.28%) and cytoplasmic (21.68%) area of the cells. Regarding the esophageal structure in general, the infection, regardless of whether treated or not, induced a reduction in the diameter of the organ, without significantly affecting the thickness of the wall and the muscular layer. From these results, the authors suggested that, in fact, ASA represents an interesting intervention to prevent atrophy of esophageal nitrergic neurons [24].

To continue evaluating the potential of ASA in experimental Chagas disease (chronic phase), Massocatto et al. [25] observed that increasing the concentration from 20 mg/kg [24] to 50 mg/kg [25] induced neuronal protection to esophageal nitrergic neurons of the myenteric plexus. However, the increase in concentration was also accompanied by neuronal atrophy, with a reduction in body areas (12.75%), nucleus (13.28%), and cytoplasm (13.03%). Interestingly, it was reported that the treatment partially prevented esophageal hypertrophy caused by the infection, by reducing the thickness of the tunica muscularis (4.33%) and of the circular muscle (11.80%) and allowing an increase of only 6.46% of increase of total organ thickness (compared to 20.37% of the untreated infected group). In addition to these parameters evaluated, the authors demonstrated that ASA improves the passage time of food through the animals' gastrointestinal tract. Thus, it is argued that ASA represents an alternative treatment for inducing nitrergic neuronal protection and reducing esophageal hypertrophy [25].

To assess the impact of ASA on the total population of colon myenteric plexus neurons, Swiss mice were infected with the Y strain of *T. cruzi* [27]. The treatment, as previously reported [24, 25], did not affect the evolution of the infection in relation to parasitemia [27]. Regarding the total number of neurons, ASA was not able to induce protection of these cells in the myenteric plexus in the distal colon, demonstrating a count similar to the reduction observed in the infected and untreated groups. In addition, the intervention induced neuronal hypertrophy with an increase in the cytoplasmic area (51.0%), nuclear (22.4%), and neuron body (39.4%) [27].

In a deeper analysis in relation to different neuronal subpopulations in the colon of Swiss mice infected with the Y strain of *T. cruzi*, Oda et al. [26] demonstrated the impact of using ASA in the acute and chronic phases. Treatment in the acute phase was able to reduce total parasitaemia, as well as the peak on different days, while treatment in the chronic phase did not change the course of infection. In addition, it was reported that the intervention was not able to change NO levels in the intestines of the animals, but rather to reduce the amount of inflammatory infiltrate in the organ, both with treatment in the acute and chronic phases. Regarding neurons, the authors demonstrated that the infection induces intense neuronal destruction, resulting in the total reduction of neurons in the myenteric plexus (60.7%), nitrergic (49%), vipergic (38%), and cholinergic (67%) subpopulations. Treatment with ASA, regardless of disease stage, reduced the destruction of all neuronal subpopulations. There was also a slight reduction in the number of these cells in uninfected treated animals. However, this reduction did not impact the animals' gastrointestinal transit. As long as the transit of the gastrointestinal tract was affected by the infection, the intervention was able to normalize the flow, regardless of the phase of exposure to ASA. The treatment was able to reduce infection-induced hypertrophy in all neuronal subpopulations only when the intervention was performed in the acute phase. In the chronic phase, it was not able to control the hypertrophy of nitrergic neurons.

Furthermore, Oda et al. [26] also evaluated the profile of neuropeptides, substance P (SP), and intestinal vasoactive peptide (VIP), involved in the pathophysiology of Chagas disease. It was shown that ASA treatment reversed the P/VIP substance profile found in *T. cruzi* infection, in which the presence of SP-containing varicosities was greater than those containing VIP. Thus, the use of ASA normalized SP levels and increased VIP levels.

## 4. Discussion

**Figure 4:**
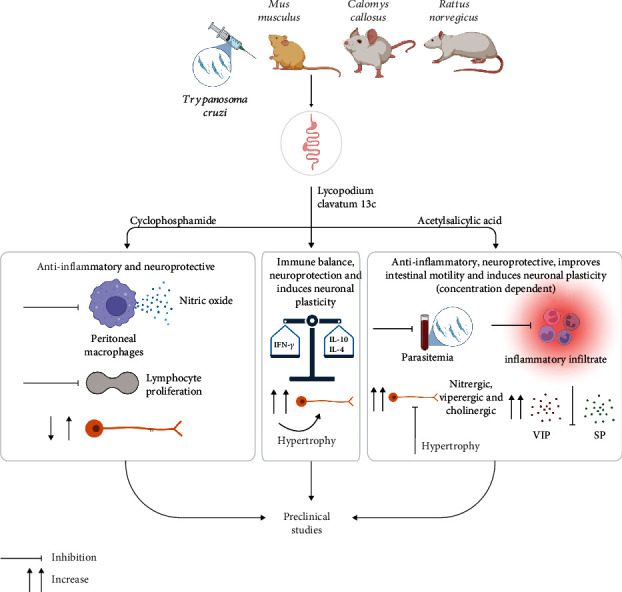
Beneficial effects of pharmacological interventions in experimental models of the digestive form of Chagas (made in ©BioRender: https://biorender.com).

The present study summarizes potential preclinical pharmacological treatments for the digestive forms of CD. Thus, it was demonstrated that only 3 interventions have evidence applied to these conditions and with beneficial effects: LC, cyclophosphamide, and ASA (Figure 4).

Thus, LC is a plant of the Lycopodiaceae family commonly associated with anti-inflammatory [28], antimicrobial, and antioxidant phenomena [29, 30]. These functions may be associated with the diverse composition of secondary metabolites found in their spores [31], such as serratan triterpenoids [32]. Besides the impact on CD, the use of LC has also been evaluated in other conditions, such as in experimental *Toxoplasma gondii* infection [33, 34], *in vitro* cytotoxic effect on colon cancer cells [35, 36], and in a pilot study in humans with irritable bowel syndrome [37].

In fact, in the work of Brustolin Aleixo et al. [20], it was observed that the use of this plant, formulated in a dynamized way, resulted in immunoregulation and, consequently, neuronal protection in mice infected with *T. cruzi*. Although only one article has evaluated the neuroprotective potential, other studies have shown that the use of CL induces protection against *T. cruzi* infection, mainly by improving clinical signs and increasing the survival of infected animals [38, 39]. The authors suggest that these findings are due to the ability of LC to induce immune homeostasis on behalf of the host. For this, it was reported that the highly diluted intervention is able to favor the Th1 profile at the beginning of the infection, at 8 [38, 39] and 10 days [20], which controls the infection by the parasite. Later (24 days), as demonstrated by Brustolin Aleixo et al. [20], there is an inversion of cytokines, favoring the control of the proinflammatory profile by anti-inflammatory (IL-4) and regulatory (IL-10) cytokines, which reduces tissue damage and is associated with the neuronal protection described. In addition to immunoregulatory activity, it was observed that LC was also able to reduce parasitaemia and amastigote nests in the heart and intestine of mice infected with *T. cruzi* in the acute phase [40]. Thus, this intervention stimulates several mechanisms of action that help the host to control the infection and consequently reduce tissue damage.

Cyclophosphamide, in turn, was used in three articles included in this systematic review [21–23]. The authors demonstrate that this intervention was able to induce neuronal protection in the colon and esophagus, as well as reduce the proinflammatory response via NO and the proliferative capacity of splenocytes [21–23]. This drug is widely known for its immunosuppressive function and marked cytotoxic effect, especially on lymphocytes [41]. This action is related to the low cellular expression of aldehyde dehydrogenase by lymphocytes, an enzyme that participates in the detoxification process of the active form of cyclophosphamide [41, 42]. In this way, these cells become more susceptible and die faster. Although no intestinal assessment related to cytokine profile, inflammatory cells, and the presence of the parasite was addressed in the studies included in this work, other studies have analyzed the effect of cyclophosphamide on the heart of animals infected with *T. cruzi* [43]. In both mice [44–46] and dogs [47], the intervention increased the myocarditis process established by the infection. However, for rats, the opposite was observed, with cyclophosphamide preventing acute myocarditis and sympathetic denervation, indicating that the inflammatory process may be one of the pathways of neuronal death [48]. The differences found in the studies may be due to the difference in the therapeutic regimen (concentration and treatment time), the experimental models used, the parasite strains, and the inoculum. Besides CD, cyclophosphamide is one of the most successful antineoplastics known today [49]. Its potential has also been described in kidney diseases [50], autoimmune rheumatic diseases [51], and dermatological diseases [52].

ASA is one of the most used drugs in the world, related to different potentials, such as antiplatelet effect, cancer prevention and treatment, prevention of preeclampsia, therapeutic potential for diabetes, and mental and neurobiological diseases [53]. It is widely known as an anti-inflammatory, mainly because it inhibits the NF-*κ*B pathway [54]. In addition, it has also been described as an inhibitor of COX1 and COX2 [55] and peripheral production of cytokines such as IL-6 and TNF-*α* [56], all proinflammatory markers. This set of anti-inflammatory actions results in the neuroprotective potential of ASA observed in experimental models of the digestive forms of CD. This drug may be related to the reduction of the inflammatory process in general in affected organs (esophagus or colon) and in a systemic way in animals, which consequently results in neuronal protection against *T. cruzi* infection [25, 26]. In fact, it has already been demonstrated that the presence of inflammatory cells such as NK cells and cytotoxic T lymphocytes, presence of the parasite (kDNA) [5, 57], and increased production of TLR8, IFN-*β* [58], TNF-*α*, and IFN-*γ* [6] by peripheral mononuclear cells are components present in individuals with digestive forms of CD and, consequently, are part of neuronal death mechanisms. This same pattern, with a proinflammatory profile and neuronal death, is also observed in experimental models, whether in the acute or chronic phases, especially in the colon [8, 9, 59–61]. Thus, it is suggested that ASA has an immunomodulatory action by favoring the inhibition of the proinflammatory and neurotoxic profile induced by the infection. However, studies focused on the immune response after ASA intervention in *T. cruzi* infection are necessary, as none of the articles included in this work focused on this point.

Interestingly, only one of the articles included in the study aimed to use ASA during the chronic phase [26]. When using the treatment in this phase, the authors observed results similar to those found when the intervention was used in the acute phase in relation to neuroprotection. Evaluating treatments in the chronic phase are extremely important, since approximately 2-27% of individuals diagnosed in the chronic phase develop digestive forms [62]. Thus, it is very important to use a drug that can reduce neuronal destruction so that it does not progress and/or can stabilize progression when the individual already has megacolon or megaesophagus. However, the evidence found in the literature focused on this aspect is rare.

In addition, it is believed that the differences found in the results of articles using ASA may be due to methodological differences for staining and subsequent neuronal counts (GIEMSA, NADPH-dp, or immunofluorescence), as well as drug concentration (20 or 50 mg/kg), route of administration (oral or intraperitoneal), therapeutic regimen, and the organ analyzed (esophagus or colon).

Besides these factors, the use of different experimental models also impacts the results obtained. Three species of animals were used in the studies included in this work: *Mus musculus* (Swiss mice), *Calomys callosus*, and *Rattus norvegicus* (Wistar lineage). The easy and practical handling, low cost, and need for low concentration of interventions in the new drug discovery phase are advantages that increase the incidence of using these experimental models in studies focused on CD [63]. With pathogenesis similar to that of CD in humans (immunological, pathological, and physiological), it is essential to consider that models such as mice and rats may not accurately reflect the progression and manifestations of CD, with dependence on the strain used in infection, concentration, route, and form of the protozoan used in inoculum and the genetic background of the experimental model [63–65]. As an example, depending on the strain, inoculum, and experimental model used, infection in the acute experimental phase can result in up to 100% mortality rate, while for humans, the rate is 5% [63, 66]. On the other hand, cardiac changes close to human chagasic heart disease are extensively reported in *T. cruzi*-infected mice and rats (cardiac fibrosis, electrocardiogram changes, inflammation, etc.) [67–74] as well as digestive changes (delayed intestinal transit time, intestinal dilatation, neuronal loss, etc.) [24–27, 75–78]. Although factors of host-parasite dynamics are related to CD progression, the studies included in this work demonstrate that the three interventions (LC, cyclophosphamide, and ASA) have neuroprotective potential, regardless of whether the model used was mouse or rat.

Neuron morphometry was also another point that showed different results between studies. When the interventions were used, three phenomena were observed: (1) induction of neuronal hypertrophy or (2) maintenance of neuronal proportions or (3) protection against the hypertrophy of these cells. These phenomena, although different, were associated with the same factors: neuroprotection and/or compensation of neuronal reduction/death caused by the infection through cellular adaptation and neuronal plasticity, in order to maintain peristalsis.

Several studies show that *T. cruzi* infection causes changes in the profile of different neuropeptides essential for the functioning of the enteric nervous system-gastrointestinal system axis, such as SP, VIP, glial fibrillary acidic protein (GFAP), morphogenetic protein type 2 (BMP2), NOS, S100, nerve growth factor (NGF), growth-associated protein 43 (GAP-43), and glial-derived neurotrophic factor (GDNF), among others [4, 8, 79–82]. These components influence and are influenced by the cellular microenvironment. The inflammatory process, for example, induced by the protozoan can alter the balance of all these systems: immune, nervous, and endocrine. Thus, developing interventions capable of inducing the balance of these systems is extremely important.

In addition, most of the included studies did not clearly report all items evaluated using the SYRCLE tool, thus making a complete analysis of methodological quality impossible. To circumvent this limitation at the level of studies, it is interesting that authors of future studies seek to describe the study methodology in more detail, to ensure better reproducibility and reliability of studies. Furthermore, it is noteworthy that due to the heterogeneity of the included studies, the meta-analysis was not tested, which is a limitation at the level of this systematic review. On the other hand, a comprehensive search, including a Latin American database, was carried out to find all articles that fit the guiding theme.

## 5. Conclusion

This systematic review addressed studies that tested possible pharmacological and neuroprotective interventions for cases of the digestive form caused by *T. cruzi* infection in murine models. Thus, three different types of therapeutic agents have been described, so far, in the literature, being acetylsalicylic acid, cyclophosphamide, and Lycopodium clavatum 13c, which showed different modes of action. Lycopodium clavatum 13c suggests an immunomodulatory activity, resulting in neuronal protection in the distal and proximal colon. Similarly, cyclophosphamide showed a neuroprotective effect in the colon, with improvement in the morphological parameters of neurons. Although it also protected esophageal neurons, these did not undergo morphological changes. On the other hand, the results of studies that evaluated acetylsalicylic acid were contradictory, as it may act as neuroprotective or neurodestructive agents. It is worth noting that acetylsalicylic acid was able to normalize the transit of the gastrointestinal tract, as well as reduce the inflammatory infiltrate in the colon in both the acute and chronic phases. Furthermore, it was able to act on the structure of the esophagus, preventing its hypertrophy. In general, it is clear that the action of treatments is dependent on different factors, including drug concentration, stage of the disease evaluated, and the region evaluated (colon or esophagus). Thus, these parameters need to be considered in future articles and comparative studies are valid to better define the magnitude of each factor in the face of interventions. Approaches to understand how these treatments influence the behavior of immune cells in the neuronal environment are also needed. Thus, it is evident that the guiding theme of this systematic review is recent and deserves more attention, since neuroprotective interventions are crucial to reduce the digestive impact caused by Chagas disease to patients.

## Figures and Tables

**Table 1:** Summary of the main data regarding interventions with neuroprotective potential in experimental models of the digestive form of Chagas disease.

Intervention	Experimental model	Evaluated groups and number of animals used	Strain	Route of infection/inoculum used	Phases	Chronic phase induction method	Treatment schedule	Intervention concentration of/treatment route	Mortality rate	Organ and region evaluated	Methodology used for neuronal analysis/analyzed region	Number of fields and neurons analyzed	Number of neurons per group	Neuronal body area per group (*μ*m^2^ or cm^2^^∗^)	Area of neuronal cytoplasm per group (*μ*m^2^)	Neuronal nucleus area per group (*μ*m^2^)	Reference
Cyclophosphamide (CY)	*Calomys callosus*	Not infected (NI) (5) Infected without treatment (IC) (5) Infected treated with cy (IC-cy) (5)	MORC-1	Intraperitoneal 1 × 10^5^	Chronic	Natural time of infection	3 consecutive days	0.2 mL of a 0.4 mg/mL solution of the drug in water/orally	Not informed	Distal esophagus	Cresyl violet staining myenteric plexus	Performed in the total area between the inner and outer muscles of the esophagus	NI: 59 ± 30.125 IC: 34 ± 19.937 IC-cy: 39 ± 9.910	NI: 28.66 ± 9.08 *μ*m^2^ IC: 29.29 ± 8.56 *μ*m^2^ IC-cy: 35.12 ± 11.54 *μ*m^2^	Unvalued	Unvalued	[22]
Lycopodium clavatum 13c (Ly)	*Rattus norvegicus*, Wistar lineage	IC (*n* = 21) Infected treated with Ly (IC-Ly) (*n* = 21)	Y	Intraperitoneal 5 × 10^6^	125 days (acute phase) 322 days (chronic phase)	Natural time of infection	2 days before infection and on days 2, 5, and 8 postinfection	10 *μ*L/mL water *ad libitum*	Not informed	Intestine proximal (PC) and distal (DC) colon	GIEMSA staining myenteric plexus	120 fields for neuron quantification 300 neurons for cell body, cytoplasm, and nucleus measurement	IC PC 125 days: 96.6 ± 18.5 IC PC 322 days: 103.7 ± 27.9 IC-Ly PC 125 days: 141.9 ± 57.0 IC-Ly PC 322 days: 107.8 ± 16.1 IC DC 125 days: 116.7 ± 13.3 IC DC 322 days: 52.9 ± 8.1 IC-Ly DC 125 days: 114.4 ± 13.0 IC-Ly DC 322 days: 93.2 ± 9.0	IC PC 125 days: 184.4 ± 135.5 IC PC 322 days: 344.4 ± 163.0 IC-Ly PC 125 days: 339.2 ± 133.0 IC-Ly PC 322 days: 403.4 ± 206.5 IC DC 125 days: 131.0 ± 108.8 IC DC 322 days: 361.9 ± 184.7 IC-Ly DC 125 days: 201.5 ± 103.9 IC-Ly DC 322 days: 358.3 ± 238.3	IC PC 125 days: 122.8 ± 102.1 IC PC 322 days: 241.0 ± 135.7 IC-Ly PC 125 days: 229.1 ± 113.0 IC-Ly PC 322 days: 291.7 ± 176.2 IC DC 125 days: 88.8 ± 79.2 IC DC 322 days: 260.2 ± 157.6 IC-Ly DC 125 days: 139.1 ± 86.4 IC-Ly DC 322 days: 256.8 ± 205.5	IC PC 125 days: 61.5 ± 42.6 IC PC 322 days: 103.4 ± 43.0 IC-Ly PC 125 days: 110.1 ± 37.6 IC-Ly PC 322 days: 111.6 ± 42.7 IC DC 125 days: 42.2 ± 35.2 IC DC 322 days: 101.6 ± 41.0 IC-Ly DC 125 days: 62.4 ± 31.2 IC-Ly DC 322 days: 101.6 ± 42.3	[20]
Acetylsalicylic acid (ASA)	Swiss mice (*Mus musculus*)	NI (*n* = 5) NI treated with ASA (NIASA) (*n* = 10) IC (*n* = 10) IC treated with ASA (ICASA) (*n* = 10)	Y	Intraperitoneal 1.300	81 days (chronic phase)	Six doses of benznidazole (Lafepe, PE, Brazil; 100 mg/kg) were administered by oral gavage 11, 13, 15, 25, 29, and 48 days after infection	Intraperitoneal consecutive days (55-63 days postinfection) and on days 65, 67, 69, 71, 73, and 75 postinfection	100 *μ*L ASA of stock solution (50 mg/kg) via not informed	Not informed	Intestine distal colon	GIEMSA staining myenteric plexus	120 neuron quantification fields 300 neurons for cell body, cytoplasm, and nucleus measurement	NI: 5819.20 ± 754.80 NI-ASA: 5415.75 ± 259.34 IC: 4769.00 ± 65.00 IC-ASA: 3987.25 ± 529.41	NI: 132.6 (96.3; 182.7) NI-ASA: 209.7 (163.7; 311.3) IC: 114.6 (82.0; 156.5) IC-ASA: 159.8 (119.0; 227.2)	NI: 75.4 (50.3; 111.6) NI-ASA: 132.0 (91.3; 227.7) IC: 65.0 (43.2; 97.8) IC-ASA: 98.2 (65.5; 153.2)	NI: 54.5 (40.3; 68.6) NI-ASA: 68.6 (52.3; 87.4) IC: 46.8 (35.4; 61.0) IC-ASA: 57.3 (43.2; 77.6)	[27]
Cyclophosphamide (cy)	*Calomys callosus*	IC (*n* = 5) Infected treated with cy (IC-cy) (*n* = 5)	MORC-1	Intraperitoneal 100.000	10 days (acute phase) 450 days (chronic phase)	Natural time of infection	Acute phase: cyclophosphamide in water from day of infection to 21 postinfection Chronic phase: 0.4 mg/mL cyclophosphamide in water 10 days before euthanasia (440 days)	Intraperitoneal acute phase: 0.2 mg/mL of Genuxal Chronic phase: 0.4 mg/mL of Genuxal	Not informed	Distal annular esophagus segments	Cresyl violet staining myenteric plexus	Total neurons count in total area between inner and outer muscle layer	IC 10 days: 23 IC 450 days: 12.6 IC-cy 10 days: 18 IC-cy 450 days: 22	IC 10 days: 64.17 ± 15.27 IC 450 days: 29.75 ± 10.89 IC-cy 10 days: 62.61 ± 18.87 IC-cy 450 days: 22.94 ± 6.60	Unvalued	Unvalued	[23]
Acetylsalicylic acid (ASA)	Swiss mice (*Mus musculus*)	NI (*n* = 10) NI treated with ASA (NIASA) (*n* = 10) IC (*n* = 10) IC treated with ASA (ICASA) (*n* = 10)	Y	Intraperitoneal 1.300	75 days (chronic phase)	Six doses of benznidazole (Lafepe, PE, Brazil; 100 mg/kg) were administered by oral gavage 11, 13, 15, 25, 29, and 48 days after infection	Treatment performed intraperitoneally daily from the 5th to the 11th day after infection	20 mg/kg	Not informed	Esophagus distal part	Nicotinamide adenine dinucleotide phosphate-diaphorase (NADPH-dp) staining myenteric plexus	100 neuron quantification fields 100 neurons for cell body, cytoplasm, and nucleus measurement	NI: 287.30 ± 7.54 NI-ASA: 304.40 ± 6.52 IC: 339.60 ± 8.24 IC-ASA: 342.10 ± 7.62	NI: 245.60 ± 4.82 NI-ASA: 221.80 ± 4.25 IC: 206.50 ± 4.22 IC-ASA: 248.40 ± 4.35	NI: 177.00 ± 4.18 NI-ASA: 153.30 ± 3.55 IC: 143.10 ± 3.80 IC-ASA: 174.10 ± 3.83	NI: 68.62 ± 1.07 NI-ASA: 68.51 ± 1.18 IC: 63.36 ± 1.12 IC-ASA: 74.29 ± 1.11	[24]
Acetylsalicylic acid (ASA)	Swiss mice (*Mus musculus*)	NI (*n* = 5) NI treated with ASA (NIASA) (*n* = 5) IC (*n* = 5) IC treated with ASA (ICASA) (*n* = 5)	Y	Intraperitoneal 1.300	75 days (chronic phase)	Six doses of benznidazole (Lafepe, PE, Brazil; 100 mg/kg) were administered by oral gavage 11, 13, 15, 25, 29, and 48 days after infection	Intraperitoneal consecutive days (55-63 days postinfection) and on days 65, 67, 69, 71, 73, and 75 postinfection	50 mg/kg	Not informed	Esophagus distal part	Nicotinamide adenine dinucleotide phosphate-diaphorase (NADPH-dp) staining myenteric plexus	100 neuron quantification fields 100 neurons for cell body, cytoplasm, and nucleus measurement	NI: 1.48 ± 0.27 NI-ASA: 1.41 ± 0.31 IC: 1.61 ± 0.06 IC-ASA: 1.61 ± 0.23	NI: 244.80 ± 4.56 NI-ASA: 249.90 ± 5.33 IC: 219.60 ± 3.95 IC-ASA: 191.60 ± 3.37	NI: 172.10 ± 3.94 NI-ASA: 178.90 ± 4.67 IC: 151.20 ± 3.31 IC-ASA: 131.50 ± 2.77	NI: 69.62 ± 1.14 NI-ASA: 68.98 ± 1.56 IC: 65.90 ± 1.15 IC-ASA: 57.15 ± 1.11	[25]
Ácido acetilsalicílico (ASA)	Swiss mice (*Mus musculus*)	NI IC NI treated with ASA in the acute phase (NIaASA) IC treated with ASA in the acute phase (ICaASA) NI treated with ASA in the chronic phase (NIcASA) IC treated with ASA in the chronic phase	Y	Intraperitoneal 1.300	75 days (chronic phase)	Six doses of benznidazole (Lafepe, PE, Brazil; 100 mg/kg) were administered by oral gavage 11, 13, 15, 25, 29, and 48 days after infection	Acute phase: treatment performed intraperitoneally daily from the 5th to the 11th day after infection Chronic phase: treatment performed intraperitoneally daily from the 55th to the 63rd day after infection. Then alternate treatment from day 65 to day 75	Acute phase: 20 mg/kg Chronic phase: 50 mg/kg	No deaths reported in any group	Intestine colon	Immunofluorescence for nNOS, VIP, SP, and myosin-V/myenteric plexus	35 fields for quantification of neurons for each marker 300 neurons for area measurement for each marker	^∗^Myosin-V: NI: 83552.63 ± 0.06 IC: 28289.47 ± 4276.32 NIa-ASA: 72039.47 ± 3618.42 ICa-ASA: 56578.95 ± 3289.47 NIc-ASA: 70394.74 ± 2631.58 ICc-ASA: 43092.11 ± 4605.26 ^∗^nNOS: NI: 12613.42 ± 447.28 IC: 6261.98 ± 492.01 NIa-ASA: 10153.35 ± 984.03 ICa-ASA: 9661.34 ± 357.83 NIc-ASA: 11316.29 ± 849.84 ICc-ASA: 9214.06 ± 357.82 VIP: NI: 1791.24 + 34.26 IC: 1097.64 + 50.01 NIa-ASA: 1757.57 + 47.79 ICa-ASA: 1548.82 + 82.05 NIc-ASA: 1198.65 + 150.34 ICc-ASA: 1703.70 + 41.26	Myosin-V: NI: 193.01 ± 7.73 IC: 250.37 ± 9.92 NIa-AAS: 142.28 ± 9.93 ICa-ASA: 201.84 ± 11.03 NIc-ASA: 178.68 ± 8.82 ICc-ASA: 210.66 ± 8.83 nNOS: NI: 146.52 ± 12.45 IC: 183.15 ± 3.66 NIa-ASA: 142.86 ± 11.44 ICa-ASA: 157.51 ± 9.52 NIc-ASA: 159.71 ± 9.52 ICc-ASA: 171.43 ± 8.79 VIP: NI: 462.04 + 19.71 IC: 554.01 + 24.09 NIa-ASA: 295.62 + 15.32 ICa-ASA: 424.81 + 24.09 NIc-ASA: 321.89 + 17.52 ICc-ASA: 486.13 + 19.7	Unvalued	Unvalued	[26]
Cyclophosphamide (cy)	*Calomys callosus*	IC (*n* = 5) Infected treated with cy (IC-cy) (*n* = 5)	MORC-1	Intraperitoneal 100.000	10 days (acute phase) 450 days (chronic phase)	Natural time of infection	Acute phase: cyclophosphamide in water from day of infection to 21 postinfection Chronic phase: 0.4 mg/mL cyclophosphamide in water 10 days before euthanasia (440 days)	Intraperitoneal acute phase: 0.2 mg/mL of Genuxal Chronic phase: 0.4 mg/mL of Genuxal	Not informed	Colon	Coloração por Cresyl violet staining Myenteric plexus	Contagem total neurons in the total area between the inner and outer muscle layer	IC 10 days: 20.3 IC 450 days: 5.2 IC-cy 10 days: 14 IC-cy 450 days: 13.5	IC 10 days: 125.59 ± 36.29 IC 450 days: 117.±47.56 IC-cy 10 days: 108.56 ± 26.49 IC-cy 450 days: 228.88 ± 111.10	Unvalued	Unvalued	[21]

## Data Availability

All the data used to support the findings of this study are included within the article and references.

## Supplementary Materials

Supplementary Materials S1: database used and search strategy used in each one. S2: screened articles and final status for inclusion in the systematic review. S3: SYRCLE tool risk of bias criteria adapted and met.
