# Supplementary material for: Neuroprotective Treatments for Digestive Forms of Chagas Disease in Experimental Models: A Systematic Review
Source: Oxid Med Cell Longev. 2022 Sep 25;2022:9397290. doi: 10.1155/2022/9397290 (PMC9527410; doi:10.1155/2022/9397290)
Supplement: Supplementary Materials — S1: database used and search strategy used in each one. S2: screened articles and final status for inclusion in the systematic review. S3: SYRCLE tool risk of bias criteria adapted and met. [file 9397290.f1.pdf]

**S1.** Data base used and search strategy used in each one.

| Data base | Search strategy |
| --- | --- |
| PubMed | #1 (((((((((("Chagas disease") OR ("Trypanosoma cruzi")) OR ("cruzi, Trypanosoma")) OR ("American Trypanosomiasis")) OR ("Trypanosomiasis, American")) OR ("Trypanosomiasis, South American")) OR ("South American Trypanosomiasis")) OR ("Trypanosoma cruzi Infection")) OR ("Infection, Trypanosoma cruzi")) OR ("Infections, Trypanosoma cruzi")) OR ("Trypanosoma cruzi Infections")) OR ("Chagas' Disease")<br>#2 Search: (Esophagus) OR (Colon)<br>#3 Search: (((((((("Myenteric plexus") OR ("Nervous system")) OR ("Plexus, Myenteric")) OR ("Auerbach's Plexus")) OR ("Auerbach Plexus")) OR ("Auerbachs Plexus")) OR ("Plexus, Auerbach's")) OR ("Nervous Systems")) OR ("System, Nervous")<br>Combination: #1 AND #2 AND #3 |
| Web of Science | #1 TS= ("Chagas disease" OR "Trypanosoma cruzi" OR "cruzi, Trypanosoma" OR "American Trypanosomiasis" OR "Trypanosomiasis, American" OR "Trypanosomiasis, South American" OR "South American Trypanosomiasis" OR "Trypanosoma cruzi Infection" OR "Infection, Trypanosoma cruzi" OR "Infections, Trypanosoma cruzi" OR "Trypanosoma cruzi Infections" OR "Chagas' Disease")<br>#2 TS= (Esophagus OR colon)<br>#3 TS= ("Myenteric plexus" OR "Plexus, Myenteric" OR "Auerbach's Plexus" OR "Auerbach Plexus" OR "Auerbachs Plexus" OR "Plexus, Auerbach's" OR "Nervous system" OR "Nervous Systems" OR "System, Nervous")<br>Combination: #1 AND #2 AND #3 |
| EMBASE | #1 ('chagas disease' OR 'trypanosoma cruzi' OR 'cruzi, trypanosoma' OR 'american trypanosomiasis' OR 'trypanosomiasis, american' |

| | |
|---|---|
| | OR 'trypanosomiasis, south american' OR 'south american trypanosomiasis' OR 'trypanosoma cruzi infection' OR 'infection, trypanosoma cruzi' OR 'infections, trypanosoma cruzi')<br>#2 esophagus OR colon<br>#3 'myenteric plexus' OR 'plexus, myenteric' OR 'auerbach plexus' OR 'auerbachs plexus' OR 'nervous system' OR 'nervous systems' OR 'system, nervous'<br>Combination: #1 AND #2 AND #3 |
| Lilacs | ("Chagas disease") OR ("Doença de Chagas") OR ("Enfermedad de Chagas") OR ("Trypanosoma cruzi") AND (Esophagus) OR (Esôfago) OR (Esófago) OR (Colon) OR (colón) AND ("Myenteric plexus") OR ("Plexo mioentérico") OR ("plexo mientérico") OR ("Nervous system") OR ("sistema nervoso") OR ("sistema nervioso") |

**S2.** Screened articles and final status for inclusion in the systematic review.

| Title | Final situation | Ref. |
|---|---|---|
| Aspirin prevents atrophy of esophageal nitrergic myenteric neurons in a mouse model of chronic Chagas disease | Included | Massocatto et al., 2015 |
| Could Cyclophosphamide exert a protective role avoiding esophagic neuron loss in Calomys callosus infected with Trypanosoma cruzi? | Included | Caetano et al., 2007 |
| Does cyclophosphamide play a protective role against neuronal loss in chronic T. cruzi infection? | Included | Caetano et al., 2008 |
| Effect of acetylsalicylic acid on total myenteric neurons in mice experimentally infected with Trypanosoma cruzi | Included | Souza et al., 2019 |
| Substance P enhances neuronal area and epithelial cell proliferation after colon denervation in rats | Excluded (Does not use an experimental model for Chagas disease) | Buttow et al., 2003 |
| Treatment with low doses of aspirin during chronic phase of experimental Chagas' disease increases oesophageal nitrergic neuronal subpopulation in mice | Included | Massocatto et al., 2017 |

| | | |
|---|---|---|
| Beneficial immunomodulatory and neuro digestive effect in Trypanosoma cruzi infection after Lycopodium clavatum 13c treatment | Included | Aleixo et al., 2017 |
| Myenteric neuroprotective role of aspirin in acute and chronic experimental infections with Trypanosoma cruzi | Included | Oda et al., 2017 |
| Influence of Trypanosoma cruzi Chronic Infection in the Depletion of Esophageal Neurons in Calomys callosus | Included | Caetano et al., 2006 |

**S3.** SYRCLE tool risk of bias criteria adapted and met.

| Criteria used for risk of bias analysis |
|---|
| 1. Was the allocation sequence adequately generated and applied?? |
| 2. Were the groups similar at baseline or were they adjusted for confounders in the analysis? |
| 3. Was the allocation to the different groups adequately concealed during? |
| 4. Were the animals randomly housed during the experiment? |
| 5. Were the caregivers and/or investigators blinded from knowledge which intervention each animal received during the experiment? |
| 6. Were animals selected at random for outcome assessment? |
| 7. Was the outcome assessor blinded? |
| 8. Were incomplete outcome data adequately addressed? |
| 9. Are reports of the study free of selective outcome reporting? |
| 10. Was the study apparently free of other problems that could result in high risk of bias?? |

Fonte: SYRCLE’s RoB tool (HOOIJMANS et al., 2014)
